# Self-management challenges of adults with type 2 diabetes mellitus in Ekurhuleni district primary health care facilities amid COVID-19 lockdown

**DOI:** 10.4102/phcfm.v16i1.4202

**Published:** 2024-04-22

**Authors:** Siphiwe S. Mahlare, Melitah M. Rasweswe, Thifhelimbilu I. Ramavhoya

**Affiliations:** 1 Department of Nursing, Faculty of Health Care Sciences, University of Pretoria, Pretoria, South Africa; 2 Department of Nursing, Faculty of Health Care Sciences, University of Limpopo, Polokwane, South Africa

**Keywords:** challenges, COVID-19, lockdown, primary health care facility, self- management, type 2 diabetes mellitus

## Abstract

**Background:**

Self-management is highly recommended in managing type 2 diabetes mellitus (T2DM). Amid the coronavirus disease 2019 (COVID-19) lockdown, many restrictions were imposed, which might have affected the continuum of care and self-management. However, little is known about how people with T2DM experienced self-management during COVID-19 lockdown within the primary health care (PHC) facilities.

**Aim:**

The study explored and described the self-management challenges of adults with T2DM in Ekurhuleni PHC facilities amid COVID-19 level 5 and 4 lockdowns.

**Setting:**

The study was conducted in three community health centres in Ekurhuleni which are rendering PHC services.

**Methods:**

A phenomenological, qualitative, exploratory, and descriptive design was utilised. Purposive sampling was used to select adult patients with T2DM. Data were collected telephonically between July 2022 and August 2022 using semi-structured interviews. Inductive content analysis was used to analyse data.

**Results:**

Two themes emerged from the interviews, namely, uncontrolled blood glucose levels and financial challenges.

**Conclusion:**

The patients with T2DM experienced uncontrolled blood glucose levels and financial challenges during the COVID-19 lockdown. Guidelines to improve self-management programmes during restrictions are needed to promote good health during future pandemics to prevent complications and mortality. The telehealth model can be designed to monitor chronic patients at home during lockdown as a two-way communication.

**Contribution:**

More knowledge and insight into self-management and health promotion of patients with T2DM was provided by this study. Increased training needs arose for PHC nurses in managing and monitoring patients.

## Introduction

Type 2 diabetes mellitus (T2DM) self-management is a recommended strategy used to control and keep blood glucose levels normal.^1^ Self-management is necessary because it assists in preventing severe complications and reducing morbidity and mortality of diabetes mellitus (DM). Self-management for T2DM includes lifestyle modifications such as reducing risk factors, healthy dietary practices, planned physical activity, monitoring of blood glucose, managing episodes of low or high blood glucose, compliance to medications, ability to resolve diabetes problems and healthy coping skills.^2^ This also includes self-management education needs, which is an ongoing, lifelong process of facilitating the knowledge and skills necessary for diabetes self-care.^3^ It is, therefore, important for the T2DM patients to adhere to the self-management behaviours to achieve the optimum glycaemic control.

To promote self-management behaviours amongst people with T2DM, nurses are expected to monitor, manage, educate and support the patients regularly, as there are identified shortcomings in implementation of self-management.^4^ The National Department of Health adult primary care guide recommends prompt and effective health promotion regarding self-management for patients with DM.^5^ Currently, in Ekurhuleni primary healthcare facilities the support is provided via face-to-face consultation to ensure good compliance, early identification of complications, and providing on-going health education.^6^ In March 2020, the World Health Organization declared the coronavirus disease 2019 (COVID-19) outbreak a global pandemic. On 15 March 2020, the South African government declared its country a State of Disaster,^7^ followed by a full lockdown, with strict regulations. The lockdown regulations were enforced for people to stay at home to curb the spread of the virus^8^ subsequently disrupting the primary health care services rendered for the patients with T2DM. The lockdown restrictions were organised in levels, level 5 started towards the end of March 2020 until end of April 2020 and was considered the most extreme. Transition to levels 4, 3, 2 and 1 occurred between May 2020 and September 2020, and only level 1, which was referred to as the ‘new normal’ lifted some restrictions, although with limitations until April 2022, except for the periods when the incidence of infections and fatalities increased, starting with the implementation of level 3 limitations from the end of December 2020 until the end of February 2021.^9^ Type 2 DM patients were reported to be less likely to use the regular services and medical care to manage diabetes during the stated lockdown restrictions.^10^ The patients encountered challenges regarding self-management and monitoring which then had an impact on compliance and control.^2^ The nurses also found it difficult to support and provide face-to-face consultation to ensure that the patients with T2DM are continuously receiving self-management health education and complying to self-management requirements.^11^

The imposed restrictions also affected the activities that are practiced in T2DM self-management programmes such as planned exercises and outdoor physical activities.^2^ As such, many T2DM patients gained weight because of binge eating, regular snacking, and limited outdoor movements, which resulted in uncontrolled blood glucose levels.^10^ The severe uncontrolled blood glucose levels resulted in many T2DM patients contracting the COVID-19 infection and had to be hospitalised in Intensive Care Units (ICU).^2,12,13^ Unsurprisingly, it was reported that adults with some underlying medical conditions, such as DM, are more likely than others to become severely ill if infected with COVID-19 or die.^14^ The case reports published by the Chinese Disease Control and Prevention Centre showed that the mortality of persons with diabetes was three times higher than that of persons without diabetes.^15^ In the Western Cape, South Africa, Dave et al.^13^ found that many DM patients were admitted for COVID-19 infection, mostly in ICU and half of the admissions resulted in death.

In Ekurhuleni, during the COVID-19 pandemic lockdown, 14% of patients with DM died because of complications that might have been caused by the imposed restrictions.^16^ During the level 4 COVID-19 lockdown, the first author, accompanied primary health care student nurses to the Ekurhuleni primary health care facilities and identified that a high number of T2DM patients are referred to the hospital with uncontrolled blood glucose levels compared to prior the pandemic. There was no literature available on the self-management challenges of the COVID-19 lockdown among patients with T2DM in Ekurhuleni. Moreover, the current study explored the self-management challenges of adults with T2DM in Ekurhuleni district primary health care facilities amid COVID-19 lockdown restrictions.

## Research methods and design

### Study design

A phenomenological, qualitative, exploratory and descriptive design was utilised in this study. The reason for using this design was to provide an in-depth understanding on lived experiences of T2DM patients’ self-management challenges during COVID-19 lockdown restrictions.

### Study setting

This study was conducted in three community healthcare centres (CHC) in the Ekurhuleni East sub-district which provides 24-h primary health care services. Ekurhuleni district is a ‘category A’ or metropolitan municipality situated at the East Rand region of Gauteng province, South Africa. This municipality executes all the functions of local government for a city. Ekurhuleni East sub-district has 28 clinics, 4 CHCs which are providing 24-h services, and 24 are local clinics which are only open for 8 h during the day.

### Study population and sampling

The study population consisted of the adult patients with T2DM at three CHC’s in Ekurhuleni East sub-district attending DM management and monitoring, because access to the fourth CHC was not granted by the local management. The non-probability purposive sampling method was used to select participants. All the T2DM patients were on one of the three CHC databases. All male and female adult patients living with T2DM and who had been receiving the primary health care services for DM in Ekurhuleni East sub-district in one of the three selected CHC’s for 2 years or more were eligible to take part in the study. The recruitment of participants was met through the three facility managers, who shared the intentions to conduct the study and the first author’s cell phone number with the adult patients who were attending T2DM follow-up services in the selected CHC’s. The patients who were interested in the study left their contact numbers with the facility manager, while others phoned or messaged the first author. The first author contacted the T2DM patients via a phone and provided them with more information about the study. Verbal consent for participation was obtained immediately after the information session. Once consent was granted, an appointment for a possible interview was made.

### Data collection

The data were collected between July 2022 and August 2022 using semi-structured interviews. The aim was to gain insight into the possible challenges of T2DM self-management experienced during level 5 and 4 COVID-19 pandemic lockdown restrictions which occurred between March 2020 and September 2020. Prior to the main study, a semi-structured telephone interview with one patient of T2DM was conducted, to check the appropriateness and sequence of the research questions, and also to improve the first author’s interview skill. The main interviews were conducted in English through a telephone and lasted between 20 and 35 min. All the interviews were audio recorded using the first author’s cell phone placed on speaker with the permission of each participant. Interview notes were written down as field notes to ensure understanding following active listening and for transcription purposes. An interview guide with relevant questions related to T2DM self-management challenges during COVID-19 lockdown restrictions was used by the first author to elicit more information from each participant. The central question was ‘How did level 5 and 4 COVID-19 pandemic lockdowns which occurred between March 2020 and September 2020, affected self-management related to your condition?’ The probing questions, such as ‘Describe how did the challenges affect your well- being and your behaviour towards your condition (type 2 diabetes mellitus)?’ were posed depending on the responses provided. Probing allowed the interviewer to diverge and pursue a response in detail, and the participants were able to elaborate and provide more information. Data were collected until data saturation was reached at participant number 15, but three more participants were added to ensure that there was no longer new information given during data collection.^17^

### Data analysis

Data analysis in this study was on-going. Thus, immediately after each telephone interview, recordings were transcribed verbatim by the first author and compared to the written notes. The recordings and transcripts were shared with co-authors and an independent co-coder who is a qualitative researcher expert for support of data coding and confirmation of the preliminary findings. The inductive content data analysis was utilised, with the aim of identifying important aspects of the content.^18^ The method followed a step-by-step process for proper organisation. The steps included were:

- Reading and reading to familiarise with the data and understanding the challenges that the adult patients with T2DM experienced amid COVID-19 lockdown restrictions.
- First round coding, whereby big picture meaning units were identified from the larger data. The participants’ own words and phrases were used to generate meaning units.
- Second round coding, in which similar words and phrases from data were identified and grouped together to form subthemes.
- The subthemes were refined by putting together similar subthemes to build up main themes. Some subthemes remained as is.
- Synthesis and interpretation of the emerged themes and subthemes were done to confirm if they answered the study questions and achieved the study objectives.

Thereafter, a meeting was held between all the authors and the independent co-coder and an agreement was reached on the themes and sub-themes that emerged from analysis.

### Trustworthiness

Trustworthiness was ensured throughout the study process using the Lincoln and Guba 1985 model.^19^ Credibility was ensured by involving the co-authors in data analysis and reviewing a manuscript draft. The independent co-coder also checked if the collected data produced similar findings as the authors. For transferability, the study was contextualised, so that participants provide extensive and detailed reports of their lived experiences in that particular setting. In addition, the adult patients with T2DM in the facilities that they receive follow-up treatment and monitoring were purposively sampled to relate the findings to their setting. Dependability was ensured by using and describing scientific research process throughout the main study. For reflexivity, data analysis was transparent enough to allow the researchers to continuously assess their own bias from the study findings, by not portraying their personal and professional opinions as the findings emerge, because they are nurses. For confirmability, all the recordings, transcripts and field notes were assigned to the co-authors and the independent co-coder to code independently and confirm the findings. Also, a clear audit trial (all research data) was properly stored electronically to facilitate the checking and validation of results.

### Ethical considerations

Ethical approval was obtained from the University, Faculty of Health Sciences Research Ethics Committee (protocol 69/2022). Permission was also obtained from the Ekurhuleni Health District to access the community health centres. The participation was voluntary; informed oral consent was obtained prior to the interview and recording. The anonymity of patients and confidentiality of their data were ensured by allocating numbers to the participants and transcripts. The data shared by the participants was accurately recorded with no false information included to comply with the *Protection of Personal Information (POPI) Act*.

## Findings

### Demographics of participants

Eighteen adult patients with T2MD were recruited, and all agreed to participate in this study. The participants included 12 females and 6 males. Their age ranged between 30 and 72 and most were in their fifties. Sixteen patients were black people and two were white people. The number of years diagnosed with T2DM ranged between 2 and 16 years. Table 1 presents the adult patients with T2DM demographics.

**TABLE 1:** Participants demographical data (*N* = 18).

Variable	Frequency
**Gender**	
Male	6
Female	12
**Race**	
Black people	16
White people	2
**Age**	
< 30	1
50–59	11
60–69	5
> 70	1
**Years diagnosed with T2DM**	
2	3
4	3
5	5
6–8	3
12	2
> 15	2

**Total**	**18**

T2DM, Type 2 diabetes mellitus.

### Self-management challenges

The self-management challenges were identified and grouped into two main themes, namely uncontrolled blood glucose levels and financial challenges. Table 2 shows the self-management challenges experienced by adult patients with T2DM at Ekurhuleni East sub-district.

**TABLE 2:** Self-management challenges of adult patients with type 2 diabetes mellitus.

Theme	Subtheme
Uncontrolled blood glucose levels	Altered dietary habits
	Limited physical activities
	Unverified information about COVID-19 infection and Diabetes Mellitus
Financial challenges	Loss of jobs
	Not having enough money to buy correct food

COVID-19, coronavirus disease 2019.

### Theme 1: Uncontrolled blood glucose levels

Uncontrolled blood glucose levels emerged as a common theme from most of the participants. The participants indicated that it was difficult for them to control and maintain the normal blood glucose levels during COVID-19 lockdown. The difficulties were related to the altered dietary changes, limited physical activities and unverified information about COVID-19 infection and DM.

#### Altered dietary habits

The findings revealed several negative highlights about the type of food and frequency of consuming food during COVID-19 lockdown restrictions. The participants knew the correct food to eat in their condition, but they mentioned that because of the COVID-19 lockdown restrictions they ate anything that was available in the house, just to fill up:

‘You know with diabetes you are supposed to eat right food like vegetables then your sugar level will be right … Instead I was eating anything that I could get in the house.’ (P9, 50, M)

Some participants indicated that before the lockdown, they avoided junk food like sweet juices but ate fresh fruits, vegetables, drinking a lot of water, but during the lockdown their diet consisted of too much starch, with fruits and vegetables, which led to increased blood sugar levels:

‘Eating right food was a big challenge as we could not go out now and again to buy the right food for us people with diabetes’, during lockdown, I was eating a lot of starch, compared to before lockdown.’ (P18, 60, F)

‘Before lockdown, I was able to avoid eating wrong food I will buy fresh fruits and vegetables, and I would drink a lot of water.’ (P14, 72, F)

#### Limited physical activities

Participants expressed concerns and frustrations by being confined to one place because of the lockdown regulations on restriction of movements. Staying at home contributed to limited physical activities and weight gain, which affected their blood glucose levels negatively:

‘During lockdown I could not even exercise because of the too much staying at home … when you have sugar diabetes, you need to do some exercise.’ (P6, 55, M)

‘During the COVID my sugar was going up and down … I did not do exercises like walking because I was always in the house.’ (P8, 62, F)

#### Unverified information about coronavirus disease 2019 infection and diabetes mellitus

The participants reported that they received a lot of unverified COVID-19 information, which made them uncertain, especially when living with DM. The uncertainties regarding how COVID-19 infection targets people living with DM negatively affected their blood glucose levels and blood pressure:

‘My sugar was going up, so is my blood pressure … I was too scared during lockdown about getting COVID-19 due to rumours that were all over regarding COVID-19 and Diabetes Mellitus.’ (P1, 54, F)

Other participants reported how the media contributed to uncertainties as some of the information was unverified and they could not differentiate between rumours and the truth. They recognised that many people with DM were easily infected with COVID-19 and died:

‘We were receiving a lot of different information from the media and not sure if is the truth or not.’ (P8, 62, F)

### Theme 2: Financial challenges

Participants recognised that their financial status contributed to the changes in their T2DM self-management amid the COVID-19 lockdown. Some of the participants’ jobs were lost because of restrictions posed during the COVID-19 lockdown; as such, patients with T2DM did not have money to buy healthy food.

#### Loss of jobs

The country was at a standstill and many businesses were not operating, as such participants did not have the income to maintain their diabetic lifestyle. Participants mentioned that this affected them because they did not have money to buy healthy food and this negatively affected their self-management for T2DM:

‘*Eish*, lockdown affected me a lot. I lost my job as I was a preschool teacher. There was no money to pay us [*a big sigh with a distressed tone*], this was a real challenge because the diabetes lifestyle is expensive.’ (P2, 55, M)

The lockdown restrictions affected the patients to earn extra income, which was used to maintain the diabetic lifestyle. Extra income was a supplement for the participants to buy food that is suitable for diabetes:

‘I could not even go out to look for piece jobs, salary from piece jobs helped me to buy the right food for my condition, because my normal salary is used for the household needs.’ (P9, 50, M)

#### Not having enough money to buy correct food

Some participants recognised money as a reason that caused challenges for them to maintain self-management. This was based on the need to buy healthy food:

‘During lockdown, I did not have enough money to buy vegetables and fruits.’ (P7, 53, F)

## Discussion

The main themes generated from the interviews revealed that the patients with T2DM experienced self-management challenges amid the COVID-19 lockdown. This finding is similar to a study which found that patients with T2DM had challenges with self-management compliance during the COVID-19 lockdown.^2^ According to Sciberrias et al.,^20^ the patients with T2DM experienced challenges and burdens to maintain healthy lifestyles as required during the COVID-19 lockdown, because there was disruption in the daily routine regarding self-management activities. Drawing from the previous authors, it was clear that the identified challenges undermined the efforts of patients with T2DM to implement the effective DM practices.

The participants in this study mentioned that it was very difficult for them to adhere or comply to activities of DM self-management as required. The identified self-management challenges were identified as uncontrolled blood glucose levels and financial challenges. The impact of lockdown would have affected their eating behaviour, which resulted with altered dietary habits. Similar to our findings, the study conducted in Switzerland on self-care and quality of life in patients with T2DM during the COVID-19 lockdown, confirmed that the patients with T2DM had changed eating habits because of limited food supply and restriction of movement during the lockdown.^21^ The restriction in food supplies during the COVID-19 lockdown might have compelled them to alter their dietary habits, which increased their blood glucose levels. Altered dietary habits include the changes of food type, preparation, mealtimes and number of meals taken per day, which contribute to poor eating habits such as an increase in carbohydrates intake and sugary food.^22^ Poor eating habits are suspected to have increased chances of COVID-19 infections in patients with T2DM, making it difficult to manage and recover them from the infection.^23^

The social distancing meant to contain and prevent the cross infection of COVID-19 would have limited the physical activities of the patients with T2DM, which resulted in boredom, increased food cravings, and frequent snacking, and subsequent weight gain. Physical activities such as household chores like preparing meals, self- care activities, engaging in their work-related activities keep people active.^24^ Therefore, not participating in household chores limited physical movements; this resulted in increased insulin resistance, increased body weight and negative effects on cardiovascular health. This caused uncontrolled blood glucose levels during the COVID-19 lockdown.^25^ According to Rastogi et al.^26^ physical activity improves fat metabolism in the muscle and helps the body to use insulin better by increasing insulin sensitivity. Hence, the physical inactivity had a negative influence on patients with T2DM self-management during the COVID-19 lockdown.^27^

It is documented that, social media outlets such as radio, television, online platforms and printed materials in general can give people awareness campaigns about health threats and precautionary measures to improve their health.^28^ The use of mass social media during the COVID-19 lockdown was found to be quick, effective and evident mediator.^29,30^ In this study, unverified information about the relationship between COVID-19 infection and DM was a challenge for the patients with T2DM. The unverified information caused uncertainties made them worried, to the extent of contributing to increased and uncontrolled blood glucose levels. The patients with T2DM in this study ideally received self-management information on maintenance of good glycaemic control from the healthcare providers. However, during the COVID-19 pandemic, they were only relying on the media, and there were uncertainties in some of the information. The study conducted on needs, concerns and self-management of people with T2DM during the COVID-19 pandemic supported the mission that patients have a need for accurate information from the healthcare providers during the pandemic.^31^ Therefore, the frequency of DM messages from healthcare providers should have been increased where knowledge was found to be inadequate.^32^ It is believed that if patients with T2DM are provided with effective information on COVID-19 and self-management, they will be responsible and manage their condition in any pandemic that may occur in the future.

Lack of finances was a challenge to some of the participants during the COVID-19 lockdown as it affects daily living and maintenance of chronic conditions like DM. A study conducted on the cost and implications of implementing the integrated chronic disease management model supports the importance of being financially stable when living with chronic conditions such as T2DM.^33^ The challenge of job losses had an impact on self-management in this study. Job losses were reported to have contributed to the financial strain on patients with T2DM and negatively affected their self-management during the COVID-19 pandemic.^34^ According to Khare and Jindal,^23^ job losses caused financial stress; this in return increased blood glucose levels in the patients with DM during the COVID-19 lockdown. Patients with T2DM rely on their jobs for stable finances to buy the correct diabetic food and to attend primary healthcare facilities for monitoring and management of their condition, to prevent the risk of developing diabetes complications.^35^

Loss of jobs contributed to a lack of money to buy the correct food suitable for patients with T2DM. A study conducted in the United States of America on food insecurity and DM concurred with the challenge of job losses, which are associated with worry of not having adequate money to buy healthy food.^36^ A study conducted on psychological, social and financial impacts of COVID-19 on culturally and linguistically diverse communities, concluded that changes in financial status, resulted in reduction in meeting family expenses for better living.^37^ Whilst these findings reiterate the pervasive financial burden for patients with T2DM to maintain healthy eating habits as self-management, financial burden has serious implications on economy and food security for South African citizens at large.

### Limitations of the study

The telephone interviews using a cell phone as a method for data collection was a challenge, as some of the participants did not honour the appointments that were agreed upon. The process of re-arranging appointments was lengthy and costly; hence, the data collection took longer. The authors could not have observed the non-verbal communication from the participants during the telephone interviews, which would have enhanced the credibility of the data collected. However, the telephone interviews ensured the participants are safe and comfortable in their own homes during data collection. The study was conducted in one sub-district, which narrowed the scope in the research; the study would have included the whole district, province, and even the country. The interviews were conducted in English only; the non-English speaking patients would have contributed more information to the study.

## Conclusion

The study explored and described the challenges of the patients regarding self-management with T2DM amid the COVID-19 lockdown. This study highlights the importance of understanding the self-management challenges amid the COVID-19 lockdown among patients with T2DM. The uncontrolled blood glucose levels because of altered dietary habits, limited physical activities and unverified information about COVID-19 infection and DM were found to have challenged the patients with T2DM self-management. Additionally, the study identified financial challenges aggravated by loss of jobs, and inadequate money to buy correct food amongst patients with T2DM amid COVID-19 lockdown.

These findings emphasise the importance to conduct research studies in other settings on the experiences of patients with T2DM and primary health care providers in management of DM patients during the COVID-19 lockdown. The training of the healthcare providers on telehealth services needs to be initiated by the Department of Health, for the promotion of continuing with healthcare including self-management and monitoring of DM at home, as a two-way communication between the healthcare provider and the patient during any pandemic that can occur in the future. Those who cannot access telehealth can be provided the opportunity to use face-to-face self-management support. There is also a need to issue and enforce the use of available guidelines on self-management programme for the patients with T2DM.
